# The contribution of plasmids to trait diversity in a soil bacterium

**DOI:** 10.1093/ismeco/ycae025

**Published:** 2024-02-14

**Authors:** Sarai S Finks, Pranav Moudgalya, Claudia Weihe, Jennifer B H Martiny

**Affiliations:** Department of Ecology and Evolutionary Biology, University of California Irvine, 321 Steinhaus Hall, Irvine, CA 92697-2525, United States; Department of Ecology and Evolutionary Biology, University of California Irvine, 321 Steinhaus Hall, Irvine, CA 92697-2525, United States; Department of Ecology and Evolutionary Biology, University of California Irvine, 321 Steinhaus Hall, Irvine, CA 92697-2525, United States; Department of Ecology and Evolutionary Biology, University of California Irvine, 321 Steinhaus Hall, Irvine, CA 92697-2525, United States

**Keywords:** microdiversity, mobile genetic elements, plasmids, genetic traits, HGT, plant litter, Curtobacterium, Actinobacteria, Actinomycetota

## Abstract

Plasmids are so closely associated with pathogens and antibiotic resistance that their potential for conferring other traits is often overlooked. Few studies consider how the full suite of traits encoded by plasmids is related to a host’s environmental adaptation, particularly for Gram-positive bacteria. To investigate the role that plasmid traits might play in microbial communities from natural ecosystems, we identified plasmids carried by isolates of *Curtobacterium* (phylum *Actinomycetota*) from a variety of soil environments. We found that plasmids were common, but not ubiquitous, in the genus and varied greatly in their size and genetic diversity. There was little evidence of phylogenetic conservation among *Curtobacterium* plasmids even for closely related bacterial strains within the same ecotype, indicating that horizontal transmission of plasmids is common. The plasmids carried a wide diversity of traits that were not a random subset of the host chromosome. Furthermore, the composition of these plasmid traits was associated with the environmental context of the host bacterium. Together, the results indicate that plasmids contribute substantially to the microdiversity of a soil bacterium and that this diversity may play a role in niche differentiation and a bacterium’s adaptation to its local environment.

A high degree of genetic variation is encompassed within traditional operational taxonomic units of bacteria [1]. This so-called microdiversity encompasses an enormous amount of variability in traits that influence a bacterium’s ecological role and its contributions to community functioning [2–4]. Plasmids may contribute to this microdiversity as they can encode a diversity of traits [5] that may allow a bacterium to adapt rapidly to environmental changes [6]. The most striking examples of this are the transfer of metal and antibiotic resistance, particularly in the human gut microbiome and clinical environments [7–10]. Beyond toxin resistance, however, evidence of the importance of plasmids to broader niche-adaptation is sporadic [11, 12]. Most of what we currently know is based on a handful of well-represented genera (e.g. *Vibrio, Pseudomonas*, and *Burkholderia*) within the phylum *Pseudomonadota* (e.g. Chibani *et al.* [13]) and few studies consider Gram-positive bacteria (e.g. Gushgari-Doyle *et al.* [14]) but see, Finks and Martiny[5].

A general understanding of plasmid evolution, the diversity of traits that they carry, and their importance for adaptation in most bacterial communities thus remains elusive [5, 15]. To investigate these unknowns in a soil bacterium, we focused on the widespread genus *Curtobacterium* [16] for which we have isolated a number of closely related strains from the top layer of soil (plant litter) in different environments. *Curtobacterium* strains associated with plant disease can carry plasmids encoding for putative virulence-encoded genes [17]. However, plasmid prevalence and diversity for this genus, as in other soil bacteria, are largely uncharacterized.

Plasmids can mobilize across broad bacterial host ranges [18], interact with other types of mobile genetic elements [19], and recombine with their hosts [20]. We thus expected that *Curtobacterium* plasmids would also be subject to a high degree of mobility and recombination. However, plasmids are also vertically transmitted to daughter cells during host cell replication such that, at some level of genetic resolution, they will be phylogenetically conserved. Thus, plasmids might be conserved within *Curtobacterium* ecotypes, previously defined as genetic clades with similar phenotypes that are adapted to local environmental conditions including temperature and moisture [21]. Alternatively, selection might act on plasmids separately from that of an ecotype’s chromosome such that plasmid traits vary by environment rather than host phylogeny. To test these alternatives, here we asked: (1) Are plasmids within the *Curtobacterium* genus phylogenetically conserved? (2) What traits do the plasmids encode and how do these compare to the chromosome? (3) Are plasmid traits correlated with the environment from which they were isolated?

Long-read sequencing of 23 strains and additional reference genomes resulted in analysis of 26 putative plasmids from 18 *Curtobacterium* strains (Fig. 1; Supplemental Methods). Three lines of evidence suggest that these sequences are indeed plasmids. First, the average plasmid guanine-cytosine (GC) content was ~7% lower relative to the host chromosomes (Fig. 1D). Second, the topology (usually circular) and replicon sizes (smaller than the chromosome) of the sequences are well-known signatures of plasmids [22, 23]. Third, all but one plasmid (pD03b) carried some kind of plasmid feature. Interestingly, two plasmids of strain P990 showed % GC contents that were half that of other plasmids (32.3% and 35.3% versus ~67%; Table S1), suggesting more recent acquisition of these mobile genetic elements. Approximately half of the plasmid sequences encoded genes for known plasmid replicon types (RepA-type, *n* = 4; Table S6) or mobilization (MOB) relaxases (MOBF or MOBP, *n* = 12; Table S7). In addition, some plasmids carried genes necessary for conjugative, cell-to-cell DNA transfer (e.g. *trwC*) and for partitioning to daughter cells during host replication and division (e.g. *parA/B/G*; Fig. S1). Based on sequencing coverage, most plasmids appeared to be present in single copies, whereas some smaller ones were present in high-copy numbers (Table S1). None of the *Curtobacterium* plasmid sequences grouped into known plasmid taxonomic units (PTUs), although this is not surprising given the low representation of *Actinomycetota* in databases (Supplementary Methods [18, 24]).

**Figure 1:**
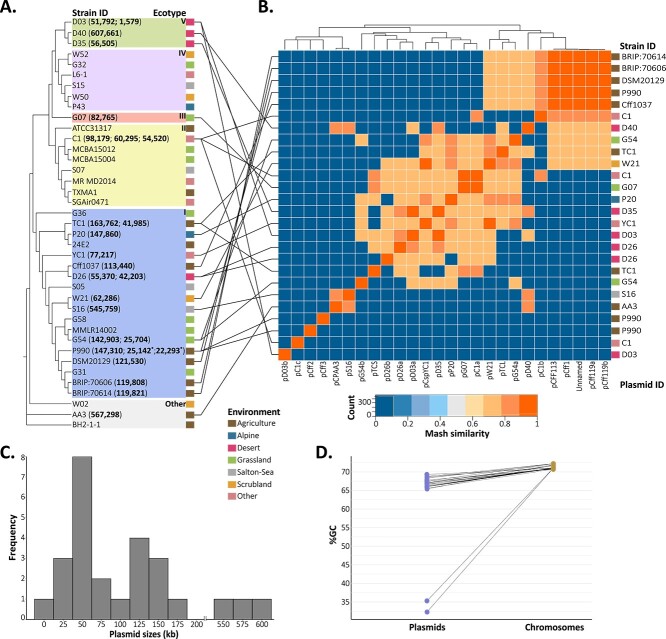
*Curtobacterium* plasmids by host ecotype and their genetic similarity, size distribution, and GC content; (A) cladogram of complete chromosomes of *Curtobacterium* constructed from a phylogenomic analysis of 916 single-copy core genes; all branches displayed represent bootstrap values of 95% confidence or greater; bolded values next to strain identifiers are nucleotide lengths of plasmids in bp; the branches are colored by ecotype designation with adjacent color tiles indicating the environment from which the strain was isolated; note: asterisks indicate the two plasmids in host P990 with relatively lower % GC content (see panel D) compared to the others; (B) heatmap of plasmids constructed from mash pairwise similarities; the strain identifiers are listed by row and the plasmid identifier (Table S1) as columns; the color tiles beside the row labels indicate the environment as in panel A; (C) the frequency of plasmid sizes in kilobases across all strains, where the *x*-axis is the lower bound of each 25kB bin; (D) percent GC content for each plasmid and its corresponding chromosome.

Plasmids were common among *Curtobacterium* strains, but their distribution across the phylogeny was not random. Plasmids were notably absent from ecotype IV and very common in ecotype I (Fig. 1A). That said, plasmid size varied greatly even within clades (1.5–607 kb, mean = 136 kb), supporting the idea that plasmids are not phylogenetically conserved in this genus (Fig. 1C). Indeed, genetic (mash) similarity of the plasmids was not correlated with the genetic similarity of the host chromosomes (Fig. 1B; RELATE: r = 0.28; *P* = 0.08).

*Curtobacterium* plasmids encoded more than 4000 gene calls that clustered into 2396 distinct orthologous groups (Fig. S1). Despite making up only 3% of the gene content of the entire dataset, this genetic diversity spanned 22 Clusters of Orthologous Genes (COG) functional categories. Based on whole genome alignments, *Curtobacterium* plasmids did not appear to share a conserved backbone, such as is commonly observed for some IncF type plasmids found in *Enterobacteriaceae* [25]. Only one gene, *lsr2* (a putative histone-like protein), was shared by 38% of the 26 plasmids, whereas most other genes were shared by fewer than three plasmids (Fig. S1). BlastP searches of consensus amino acid sequence alignments of Lsr2 against the National Center for Biotechnology Information (NCBI) Reference Proteins (refseq_protein) database reveals that this small protein (~12 kDa) is ubiquitous throughout the genus. In *Mycobacterium smegmatis,* this protein appears to be involved in the biosynthesis of mycolyl-diacylglycerols, an apolar lipid in the cell wall, as well as a DNA-binding function having a transcriptional regulatory role [26–28].

The *Curtobacterium* plasmids encoded a diversity of traits that were not a random subset of chromosomal traits (*G* (21) = 1203.2, *P* < 0.001; Fig. 2A). Not surprisingly, genes associated with the mobilome, prophages, and transposons (X) were relatively more prevalent on plasmids than the chromosome, but other functions including those associated with cell motility (N) were relatively more abundant on plasmids than on chromosomes (Fig. 2B). Conversely, carbohydrate transport and metabolism functions (G) were more prevalent on *Curtobacterium* chromosomes than plasmids. Given their role in soil carbon cycling, it is notable that 11 plasmids carried 46 CAZyme (carbohydrate active enzyme) genes (Table S9; Fig. S2A), and in more than half of these cases, the CAZyme family was not present on the associated host chromosome. We also identified two genes encoding nitrate assimilation (*narB*) on a plasmid (Fig. S2B).

**Figure 2:**
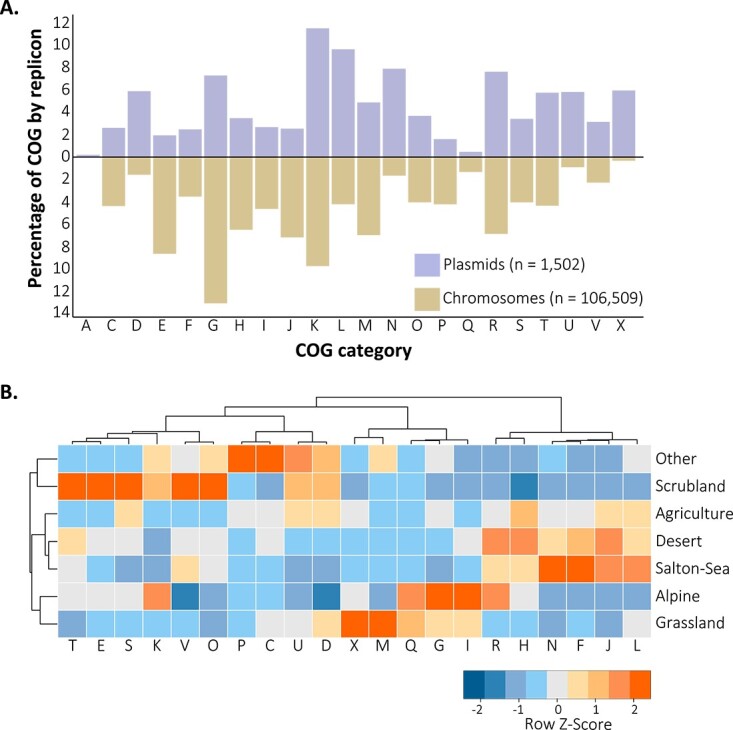
*Curtobacterium* plasmid COG functions are distinct from chromosomal functions and vary by environment; (A) percentages (Log_10_ scaled) of COG functional category counts of the *Curtobacterium* plasmids (top) and chromosome (bottom) sequences; the total number of COG functions identified on *Curtobacterium* plasmids and chromosomes are shown in parentheses; no COG functions for category Z were identified on the plasmids, and plasmids pCff2, pCff3, and pD03b are not included as no COG functions were identified; (B) normalized frequencies of COG categories encoded by the plasmids by environment; the counts of COG functions were first converted into proportional abundances within an environment after removal of COG functions (*n* = < 6), and COG counts were then normalized across environments using Z-scores to standardize for uneven representation of plasmids across environments; C—energy production and conversion; D—cell cycle control, cell division, chromosome partitioning; E—amino acid transport and metabolism; F—nucleotide transport and metabolism; G—carbohydrate transport and metabolism; H—coenzyme transport and metabolism; I—lipid transport and metabolism; J—translation, ribosomal structure and biogenesis; K—transcription; L—replication, recombination and repair; M—cell wall/membrane/envelope biogenesis; N—cell motility; O—post-translational modification, protein turnover, chaperones; P—inorganic ion transport and metabolism; Q—secondary metabolites biosynthesis, transport and catabolism; R—general function; S—unknown function; T—signal transduction mechanisms; U—intracellular trafficking, secretion, and vesicular transport; V—defense mechanisms; X—mobilome: prophages, transposons.

Finally, plasmid trait composition differed significantly by the environment from which the host was isolated, explaining ~14% of variation in COG functional categories (PERMANOVA: Pseudo-F (7): 1.424, *P* = 0.042). For instance, plasmids isolated from grassland and alpine environments encoded a higher prevalence of carbohydrate transport and metabolism (G) genes, whereas those isolated from two arid environments (Desert and Salton-Sea) encoded a relatively high number of genes associated with cell motility (N) and translation, ribosomal structure, and biogenesis (J) (Fig. 2B).

Our results indicate that plasmids contribute substantially to the microdiversity of *Curtobacterium* and that this diversity may play a role in its adaptation to the local environment. Horizontal transfer appeared to break up any signal of vertical transmission of plasmids, even within *Curtobacterium* ecotypes. However, only about half the plasmids encoded for genes known to facilitate mobility from one bacterium to another. This result is similar to that of marine *Vibrio* spp., where plasmids also appear to spread rapidly by horizontal gene transfer, many by unknown mechanisms [29].

This work also highlights the paucity in knowledge about which plasmid traits will be favored in natural ecosystems. Models investigating the evolutionary mechanisms that sustain plasmid diversity suggest that they should encode traits, like antibiotic resistance, that are widely beneficial to many bacterial species and come under relatively strong selection [30]. Future investigations into how, when, and where plasmid traits such as cell motility provide soil bacteria with an advantage would provide a more in-depth understanding of the eco-evolutionary role of these mobile genetic elements in soil.

## Supplementary Material

Finks_etal_2024_figS1_S2_final_ycae025

Finks_etal_2024_r1_ycae025

Finks_etal_supplemental_methods_r3_ycae025

## Data Availability

All scripts are accessible on GitHub (https://github.com/SaraiFinks). The ONT sequenced *Curtobacterium* and assemblies can be retrieved from NCBI SRA and Genome databases under BioProject ID PRJNA391502.
