# Supplementary material for: The contribution of plasmids to trait diversity in a soil bacterium
Source: ISME Commun. 2024 Feb 14;4(1):ycae025. doi: 10.1093/ismeco/ycae025 (PMC10999282; doi:10.1093/ismeco/ycae025)
Supplement: Finks_etal_2024_figS1_S2_final_ycae025 [file finks_etal_2024_figs1_s2_final_ycae025.pdf]

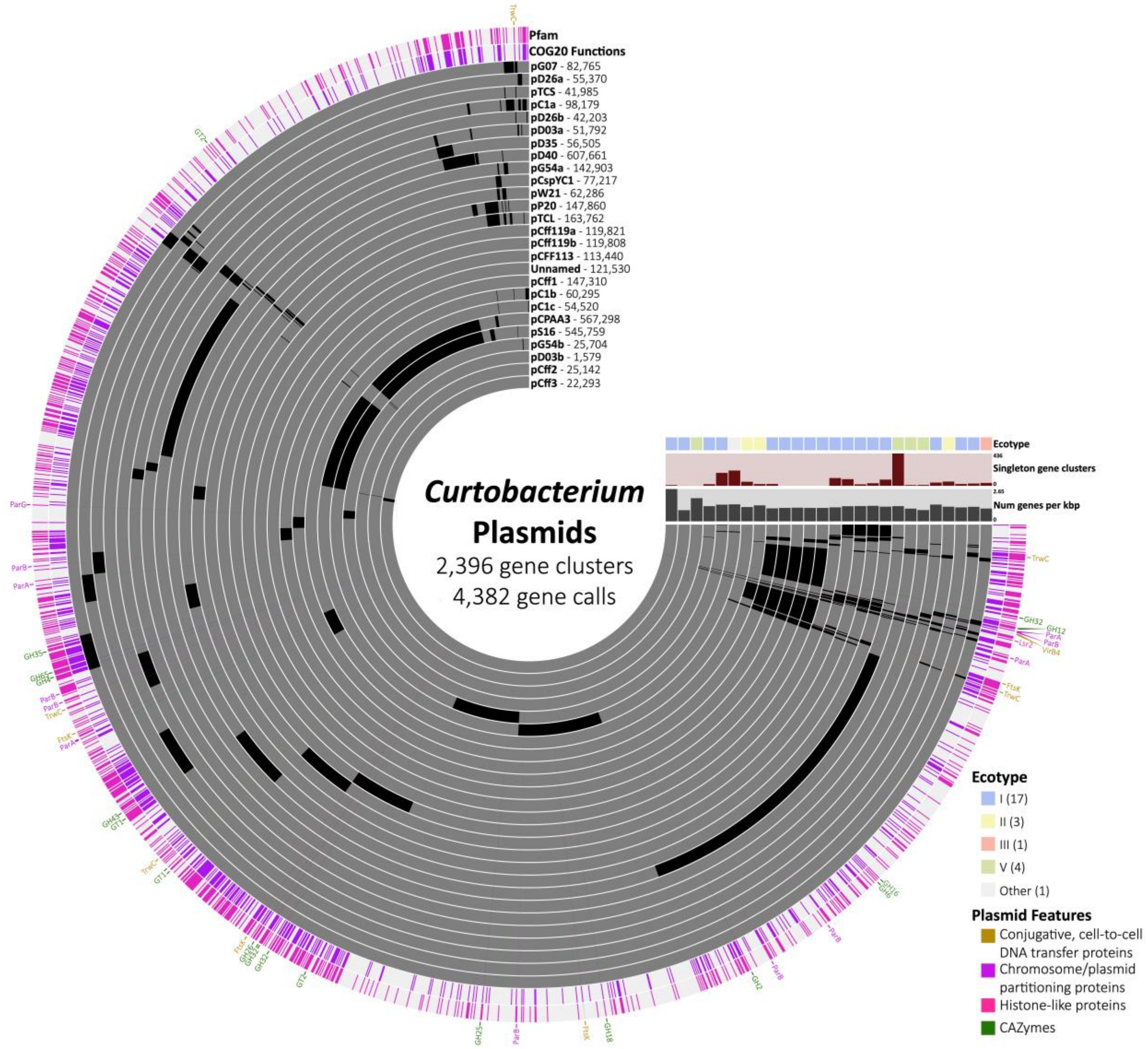

Figure S1. *Curtobacterium* plasmidome analysis. A visualization of 26 circularized/complete plasmids (rings) representing four host ecotypes and sizes ranging from approximately 1.5 – 607 kbp (nucleotide length in bp to right of plasmid ID). Common plasmid features for replication/separation during host cell division, carbohydrate utilization, and DNA protection are denoted by gene names around the outer ring. Pfam (pink) and COG20 functions (purple) that were identified are displayed by colored bars. Gene clusters are represented by black bars within gray rings and organized by presence/absence and Euclidean distance using Ward linkage (see **Supplementary Methods**).

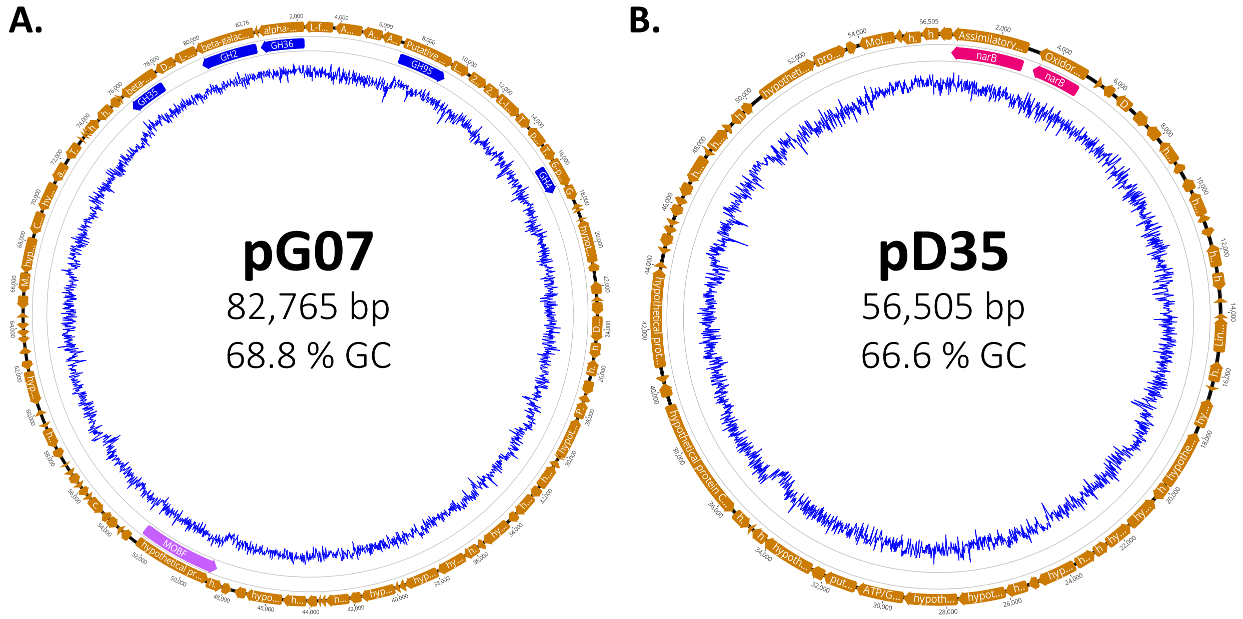

Figure S2. Schematic representation of two putative *Curtobacterium* plasmids assembled from Grassland-G07 (**A**) and Desert-D35 (**B**) strains (see **Table S1**). The putative CAZymes identified in pG07 are glycoside hydrolases GH2,4,35,36,95 shown with blue arrows. The putative assimilatory nitrate reductases, encoded by *narB* are shown with pink arrows in the pD35.
