# Supplementary material for: The contribution of plasmids to trait diversity in a soil bacterium
Source: ISME Commun. 2024 Feb 14;4(1):ycae025. doi: 10.1093/ismeco/ycae025 (PMC10999282; doi:10.1093/ismeco/ycae025)
Supplement: Finks_etal_supplemental_methods_r3_ycae025 [file finks_etal_supplemental_methods_r3_ycae025.docx]

**SUPPLEMENTAL METHODS**

**Culture collection and reference genomes.** We long-read sequenced 23 *Curtobacterium* strains from our culture collection that were obtained from senescent plant litter (the top 0-5 cm of soil) along an elevation gradient in Southern California. The strains were stored in 25% *v*/*v* glycerol at -80 ºC and had been previously sequenced on an Illumina platform [1–3]. In addition, we retrieved 14 complete plasmid sequences (and associated host chromosomes) representing diverse *Curtobacterium spp*. hosts that were deposited in NCBI GenBank and RefSeq databases on March 31, 2022. The search criteria we used included: ‘*Curtobacterium’* and ‘Plasmid’ or ‘Chromosome’. In total, we include 39 *Curtobacterium* genomes in our analyses (**Table S1**). Notably, several attempts were made to isolate plasmids from several strains in our culture collection using Qiagen® Plasmid Maxi Kit (Qiagen, Hilden, Germany) and ZymoPURE™ II Plasmid Midiprep Kit (Zymo Research, Irvine, CA, USA), and custom protocols designed to extract plasmid DNA from gram-positive taxa. However, these approaches missed many of the plasmids that the long read sequencing revealed, presumably because larger plasmids and those with low copy numbers can evade detection with traditional approaches.

**DNA preparation and sequencing**. Cultures were revived from glycerol stocks, and DNA extractions performed using the Qiagen Blood and Cell Culture DNA Mini Kit (Qiagen, Hilden, Germany). The DNA extraction generated high molecular weight gDNA (> 60 ng), free of small DNA contamination, which was suited for sequencing on Oxford Nanopore Technologies (ONT) platform. DNA quality was assessed via Nanodrop (Thermo Fisher; Massachusetts, USA) and quantified by Qubit (BioTek; Vermont, USA). ONT sequencing libraries were prepared with Ligation Sequencing Kit V14 with Native barcoding (Oxford Nanopore Technologies; Oxford, UK), multiplexed, and run on three different MinION devices with R9.4.1 flow cells by The SeqCenter Team, formerly known as The Microbial Genome Sequencing Center (Pennsylvania, USA), generating 300 Mbp per isolate. Basecalls of the raw nanopore reads were performed using Guppy v5.0.16.

**Sequence assemblies.** *De novo* ‘hybrid’ assemblies of ONT and Illumina sequenced *Curtobacterium* strains were performed with quality checked short and long reads using the default settings of Unicycler v0.4.8 [4]. Prior to assembly, quality checks for both ONT and Illumina sequencing data were checked using FastQC v0.11.9 and reports compiled using MultiQC v1.9 [5]. For ONT generated reads, low quality (PRED < 8), adaptor, and chimeric sequences were removed using Porechop v0.2.4 along with sequences < 2 kbp in length per previously described methods [6]. For Illumina generated reads, low quality (PHRED < 30), adapter, and PhiX sequences were removed using FastP v0.20.0 [7]. The read quality for both ONT and Illumina quality filtered reads were reassessed with FastQC and MultiQC. A ‘hybrid’ assembly (combining long and short read sequencing data) approach was used to obtain complete replicon assemblies, as many long reads can exceed the length of repeats in bacterial genomes, which are also a characteristic of many types of MGE, and short reads can improve accuracy of detecting plasmids in WGS data [6]. Notably, for the Scrubland-52 (W52) and Pine-Oak-43 (P43) genomes, these hybrid assemblies failed, and long-read only assemblies using Trycycler v0.5.3 was performed along with a final polishing step using Medaka v1.6.0 [8]. All assembly graphs were assessed using Bandage v0.8.1 [9], and completeness of genome assemblies (e.g., contiguity, N50, and %GC) determined using the web interface of Quast [10].

**Phylogenomic analysis***.* To determine the similarity of NCBI retrieved plasmid and chromosomes sequences to previously described ecotypes (genetic clades with similar phenotypes that are adapted to local environmental conditions including temperature and moisture) of *Curtobacterium* [1] from our culture collection, reference sequences were imported into Anvi’o v7.0 [11]. First, 916 single-copy core genes within chromosomes sequences were identified, concatenated, and nucleotide positions that were gap characters in more than 50% of the sequences removed using trimAl v1.4.1. Next, IQ-TREE [12, 13] with the ‘WAG’ [14] general matrix model was used to construct a maximum likelihood tree, which was visualized using iTOL v5 [15]. Except for three strains (AA3, BH2-1-1 and W02), the *Curtobacterium* strains in this study fell within five previously described ecotypes (based on clade designations).

Putative plasmids were identified as closed, circular sequences that were distinct from the chromosome (those having similar percent GC content to known *Curtobacterium* plasmids). No genes were conserved across all plasmid sequences, and the nucleotide lengths of putative plasmids varied significantly. Therefore, pairwise estimates of plasmid similarities were calculated using Mash v2.3 [16, 17]. The parameters for calculating mash distances were as follows: K-mer = 21 and minimum-hashes per sketch = 1000 (**Table S2** and **S3**). This comparison method was chosen because it allows for the similarity of the original sequences to be rapidly estimated with a bounded error. It depends only on the size of the sketch (i.e., the mash similarities are independent of the genome sizes) and is strongly correlated with ANI [16]. Mash distances for chromosomal sequences were also calculated using the same approach as for plasmids (**Table S2**). To evaluate whether putative plasmids of *Curtobacterium* grouped into known plasmid taxonomic units (380 PTUs constructed from 9,894 plasmid sequences from a curated reference database - RefSeq84), the web version of COPLA was used [18]. To investigate whether any of the plasmids shared conserved backbone region as is common with other types of plasmids [19], whole genome alignments were performed using Mauve v1.1.3 [20] with a seed weight set to 15 and minimum LCB score of 30,000.

**Trait analyses***.* To determine the trait content of chromosomes and plasmids, gene calls were made in Anvi’o using Prodigal v2.6.3 [21] and searched against the COG20 (Clusters of Orthologous Groups of genes/proteins) [22] and Pfam v33.1 [23] databases via DIAMOND v0.9.14 [24] in sensitive mode (**Tables S4** and **S5**). Putative plasmid replicases (used in plasmid replicon typing/incompatibility grouping) were identified from hits to the Pfam databases (**Table S6**). Clustering analysis of plasmid and chromosome amino acid sequence similarities were performed in Anvi’o using the MCL algorithm [25], under the following parameters: exclude partial gene calls, minimum gene cluster occurrence = 1, and default settings for minbit heuristic and MCL inflation parameter. Gene clusters for plasmid and chromosome replicons, visualized via the anvio-display-pan feature of the interactive interface. All COG functions, Pfam hits, and corresponding gene calls were exported as tables from Anvi’o and merged into one data table before importing into R v4.2.2 [26] for statistical analysis. To determine the potential for plasmids to be mobilizable, sequences were searched for MOB family relaxases, enzymes essential for conjugative DNA processing [27] using MobScan (**Table S7)** [28].

Additionally, chromosome and plasmid sequences were analyzed for genes involved in carbohydrate and nitrogen utilization. To identify carbohydrate active enzymes (CAZymes), we used run_dbcan v4.0.0 and dbCAN2 databases released in 2022 [29]. Query matches were included if two or more of the three search tools (HMMER, DIAMOND, Hotpep) identified the same CAZyme family annotation per the developer’s recommendation [29]. Query results were included in analyses for HMMER searches of dbCAN and dbCAN-sub with E-values < 1e-15 and coverage > 0.35; and for DIAMOND searches of the CAZy database with E-value < 1e-102 (**Tables S8** and **S9**). To identify genes associated with nitrogen-cycling pathways, BLASTp searches of queries against a curated database of nitrogen (N) gene families, the NCycDB release 2019 [30] at 100% sequence identity were performed and gene calls having E-values 10^-5^ and > 50 % query coverages were included in the analyses (**Tables S10)**.

**Statistical analysis**. To determine whether the pairwise similarities for plasmid and chromosome sequences varied by ecotype and/or environment type, similarity matrices for each sequence type were tested separately via permutational multivariate analysis of variance (PERMANOVA; permutations *n* = 999 with unrestricted permutations of raw data using type III sums of squares) in PRIMER-e v6 [31, 32] with ecotype and/or environment designated as fixed factors. Distance-based tests for homogeneity of multivariate dispersions were also performed using the PERMDISP function in PRIMER-e, grouping by either ecotype or environment. To account for sampling biases for rare ecotypes (i.e., *Curtobacterium* chromosomes outside ecotype/clade I or V; **Table S1**) and environments (i.e., *Curtobacterium* isolated from algae or unknown origins; **Table S1**), the number of plasmids/chromosomes by category were grouped together into an ‘Other’ category. The estimated variance explained was determined by dividing terms with significant p-values plus the residual variation by the sum of the estimates of components of variation given as output from PRIMER-e. To test whether plasmids and chromosome genetic similarities varied similarly by ecotype and environment, a RELATE test [32] using Spearman correlation was performed in PRIMER-e.

To determine whether the COG and CAZyme composition of plasmid and chromosomes varied by ecotype and/or environment type, euclidean distances were calculated from COG and CAZyme counts using the *vegdist* function of the ‘vegan’ package in R [33], and PERMANOVA and RELATE tests performed as previously mentioned. Heatmaps for plasmid pairwise similarities and COG traits were passed to the *heatmap.2* function of the ‘gplots’ package (https://github.com/talgalili/gplots) in R for visualization. Plasmid sequences and alignments were visualized and annotated in Geneious Prime® 2023.2.1, Build 2023-07-20 (https://www.geneious.com). Additional, G-tests were performed on contingency tables of non-standardize trait counts with rare traits (traits counts < 6 across all environments) removed to confirm trends were not stochastic attributes of these sequences.

**REFERENCES**

1. Chase AB, Gomez-Lunar Z, Lopez AE, Li J, Allison SD, Martiny AC, et al. Emergence of soil bacterial ecotypes along a climate gradient. *Environ Microbiol* 2018; **20**: 4112–4126.

2. Glassman SI, Weihe C, Li J, Albright MBN, Looby CI, Martiny AC, et al. Decomposition responses to climate depend on microbial community composition. *Proceedings of the National Academy of Sciences* 2018; **115**: 11994 LP – 11999.

3. Glassman SI, Martiny JBH. Broadscale Ecological Patterns Are Robust to Use of Exact Sequence Variants versus Operational Taxonomic Units. *mSphere* 2018; **3**: e00148-18.

4. Wick RR, Judd LM, Gorrie CL, Holt KE. Unicycler: Resolving bacterial genome assemblies from short and long sequencing reads. *PLoS Comput Biol* 2017; **13**: e1005595.

5. Ewels P, Magnusson M, Lundin S, Käller M. MultiQC: summarize analysis results for multiple tools and samples in a single report. *Bioinformatics* 2016; **32**: 3047–3048.

6. Wick RR, Judd LM, Gorrie CL, Holt KE. Completing bacterial genome assemblies with multiplex MinION sequencing. *Microb Genom* 2017; **3**: e000132.

7. Chen S, Zhou Y, Chen Y, Gu J. fastp: an ultra-fast all-in-one FASTQ preprocessor. *Bioinformatics* 2018; **34**: i884–i890.

8. Wick RR, Judd LM, Cerdeira LT, Hawkey J, Méric G, Vezina B, et al. Trycycler: consensus long-read assemblies for bacterial genomes. *Genome Biol* 2021; **22**: 1–17.

9. Wick RR, Schultz MB, Zobel J, Holt KE. Bandage: interactive visualization of de novo genome assemblies. *Bioinformatics* 2015; **31**: 3350–3352.

10. Gurevich A, Saveliev V, Vyahhi N, Tesler G. QUAST: quality assessment tool for genome assemblies. *Bioinformatics* 2013; **29**: 1072–1075.

11. Eren AM, Kiefl E, Shaiber A, Veseli I, Miller SE, Schechter MS, et al. Community-led, integrated, reproducible multi-omics with anvi’o. *Nat Microbiol* 2021; **6**: 3–6.

12. Nguyen L-T, Schmidt HA, von Haeseler A, Minh BQ. IQ-TREE: A Fast and Effective Stochastic Algorithm for Estimating Maximum-Likelihood Phylogenies. *Mol Biol Evol* 2015; **32**: 268–274.

13. Minh BQ, Schmidt HA, Chernomor O, Schrempf D, Woodhams MD, von Haeseler A, et al. IQ-TREE 2: New Models and Efficient Methods for Phylogenetic Inference in the Genomic Era. *Mol Biol Evol* 2020; **37**: 1530–1534.

14. Whelan S, Goldman N. A General Empirical Model of Protein Evolution Derived from Multiple Protein Families Using a Maximum-Likelihood Approach. *Mol Biol Evol* 2001; **18**: 691–699.

15. Letunic I, Bork P. Interactive Tree Of Life (iTOL) v5: an online tool for phylogenetic tree display and annotation. *Nucleic Acids Res* 2021; **49**: W293–W296.

16. Ondov BD, Treangen TJ, Melsted P, Mallonee AB, Bergman NH, Koren S, et al. Mash: Fast genome and metagenome distance estimation using MinHash. *Genome Biol* 2016; **17**: 1–14.

17. Ondov BD, Starrett GJ, Sappington A, Kostic A, Koren S, Buck CB, et al. Mash Screen: High-throughput sequence containment estimation for genome discovery. *Genome Biol* 2019; **20**: 1–13.

18. Redondo-Salvo S, Bartomeus-Peñalver R, Vielva L, Tagg KA, Webb HE, Fernández-López R, et al. COPLA, a taxonomic classifier of plasmids. *BMC Bioinformatics* 2021; **22**: 1–9.

19. Fernandez-Lopez R, de Toro M, Moncalian G, Garcillan-Barcia MP, de la Cruz F. Comparative genomics of the conjugation region of F-like plasmids: Five shades of F. *Front Mol Biosci* 2016; **3**: 71.

20. Darling ACE, Mau B, Blattner FR, Perna NT. Mauve: Multiple Alignment of Conserved Genomic Sequence With Rearrangements. *Genome Res* 2004; **14**: 1394–1403.

21. Hyatt D, Chen G-L, LoCascio PF, Land ML, Larimer FW, Hauser LJ. Prodigal: prokaryotic gene recognition and translation initiation site identification. *BMC Bioinformatics* 2010; **11**: 119.

22. Galperin MY, Wolf YI, Makarova KS, Vera Alvarez R, Landsman D, Koonin E V. COG database update: focus on microbial diversity, model organisms, and widespread pathogens. *Nucleic Acids Res* 2021; **49**: D274–D281.

23. Mistry J, Chuguransky S, Williams L, Qureshi M, Salazar GA, Sonnhammer ELL, et al. Pfam: The protein families database in 2021. *Nucleic Acids Res* 2021; **49**: D412–D419.

24. Buchfink B, Xie C, Huson DH. Fast and sensitive protein alignment using DIAMOND. *Nat Methods* 2015; **12**: 59–60.

25. van Dongen S, Abreu-Goodger C. Using MCL to Extract Clusters from Networks BT  - Bacterial Molecular Networks: Methods and Protocols. In: van Helden J, Toussaint A, Thieffry D (eds).2012. Springer New York, New York, NY, pp 281–295.

26. R Core Team (2020). R: A language and environment for statistical computing. *R: A language and environment for statistical computing R Foundation for Statistical Computing, Vienna, Austria* . 2020.

27. Garcillán-Barcia MP, Francia MV, de La Cruz F. The diversity of conjugative relaxases and its application in plasmid classification. *FEMS Microbiol Rev* 2009; **33**: 657–687.

28. Garcillán-Barcia MP, Redondo-Salvo S, Vielva L, de la Cruz F. MOBscan: Automated Annotation of MOB Relaxases. *Methods Mol Biol* 2020; **2075**: 295–308.

29. Zheng J, Ge Q, Yan Y, Zhang X, Huang L, Yin Y. dbCAN3: automated carbohydrate-active enzyme and substrate annotation. *Nucleic Acids Res* 2023; **51**: W115–W121.

30. Tu Q, Lin L, Cheng L, Deng Y, He Z. NCycDB: a curated integrative database for fast and accurate metagenomic profiling of nitrogen cycling genes. *Bioinformatics* 2019; **35**: 1040–1048.

31. Clarke KR, Gorley RN. PRIMER v6: *Primer V6: User Manual/Tutorial* . 2006.

32. Anderson MJ, Gorley RN, Clarke KR. PERMANOVA+ for PRIMER: Guide to Software and Statistical Methods. *Plymouth, UK*. 2008.

33. Oksanen J, Blanchet FG, Friendly M, Kindt R, Legendre P, McGlinn D, et al. vegan: Community Ecology Package. R package version 2.5-2. *Cran R* 2019.
